# Sex differences in heart: from basics to clinics

**DOI:** 10.1186/s40001-022-00880-z

**Published:** 2022-11-09

**Authors:** Chandra Prajapati, Jussi Koivumäki, Mari Pekkanen-Mattila, Katriina Aalto-Setälä

**Affiliations:** 1 Faculty of Medicine and Health Technology, Tampere University, Arvo Ylpön Katu 34, 33520 Tampere, Finland; 2 Heart Center, Tampere University Hospital, Ensitie 4, 33520 Tampere, Finland

**Keywords:** Sex differences, Electrocardiogram, Arrhythmias, Heart failure, Torsades de pointes, iPSC modeling, Animal experiments

## Abstract

Sex differences exist in the structure and function of human heart. The patterns of ventricular repolarization in normal electrocardiograms (ECG) differ in men and women: men ECG pattern displays higher T-wave amplitude and increased ST angle. Generally, women have longer QT duration because of reduced repolarization reserve, and thus, women are more susceptible for the occurrence of torsades de pointes associated with drugs prolonging ventricular repolarization. Sex differences are also observed in the prevalence, penetrance and symptom severity, and also in the prognosis of cardiovascular disease. Generally, women live longer, have less clinical symptoms of cardiac diseases, and later onset of symptoms than men. Sex hormones also play an important role in regulating ventricular repolarization, suggesting that hormones directly influence various cellular functions and adrenergic regulation. From the clinical perspective, sex-based differences in heart physiology are widely recognized, but in daily practice, cardiac diseases are often underdiagnosed and untreated in the women. The underlying mechanisms of sex differences are, however, poorly understood. Here, we summarize sex-dependent differences in normal cardiac physiology, role of sex hormones, and differences in drug responses. Furthermore, we also discuss the importance of human induced pluripotent stem cell-derived cardiomyocytes in further understanding the mechanism of differences in women and men.

## Highlights

- Sex differences exist from the cardiac structure and function to the presentation and progression of cardiac diseases.
- After puberty, QT_C_ difference between boy and girls visible, but diminish with age and disappears after 75 years of age.
- Sex hormones play an important role in the sex-specific differences mainly by affecting cardiac repolarization
- Women have a higher risk of developing a special type of ventricular tachycardia associated with adverse effects of drugs that prolong ventricular repolarization time.
- HiPSC-CMs offer a robust human-based platform for studying sex-specific drug responses and for studying the mechanisms of sex differences at cellular and tissue levels.

## Introduction

Men and women have similar genetic materials except the amount and presence of sex chromosomes, but they differ in cardiac anatomy and physiology. However, sex-specific differences extend from cardiac structure and function to the presentation and progression of cardiac diseases, as recently reviewed by Shufelt et al. [1]. Men are generally more susceptible and at earlier age to cardiovascular diseases, such as coronary artery disease than women [2]. Typically, men develop heart failure (HF) with reduced ejection fraction, whereas women develop HF with preserved ejection fraction [3], and, men develop atrial fibrillation approximately 10 years earlier than women [4]. Furthermore, right ventricular outflow tract tachycardia has sex-specific triggers; for instance, arrhythmia is initiated by hormonal fluxes in women and exercise and/or stress in men [5]. Even though the age at diagnosis is significantly lower in men, women are usually more symptomatic at the time of diagnosis [6]. The most severe clinical manifestations, i.e., ventricular fibrillation or sudden cardiac death are 8–10 times more prevalent in men than in women [7], indicating that women have better outcomes for cardiovascular major events [8].

Although endogenous sex hormones play an important role in cardiac physiology, detailed reports on the mechanisms behind these sex differences remain unavailable. From the electrophysiological point of view, women exhibit higher beating rates and slower repolarization than men. The underlying differences have been observed at the level of individual cardiomyocytes (CMs). This has led to the comprehensive research interest in identifying the cellular mechanisms responsible for sex differences in CM function. Furthermore, men undergo greater cardiac remodeling with aging than women. However, owing to limited human-based research material, most of the studies identifying the key mechanisms underlying the sex differences are conducted in animals. Although these animal studies have depicted numerous meaningful reasons behind sex differences, their outcomes cannot be extrapolated directly to humans because of fundamental differences in animal and human cardiac physiology and estrous cycle [9]. In addition, studies about sex differences have many confounding variables that might affect the results such as diet, exercise, and environment. Most of these problems can be solved with the recent progress in human induced pluripotent stem cell-derived CMs (hiPSC-CMs) [10], which are not only human-based but also enable conducting research on sex difference in a controlled environment.

This review aims to provide an overview of the currently known sex-specific differences in the cardiac electrophysiology at the organ and cellular levels. Additionally, the effect of sex hormones on ion channels is discussed as well as sex-specific differences in cardiovascular drug responses.

## Normal heart and age

The normal structure and physiology of the heart differ in men and women, and these differences reach their peak after puberty.

### Size and structure 

For men and women, the left ventricular (LV) mass values are similar during infancy and childhood, suggesting an almost the same initial number of CMs [11]. The clear sex difference in LV mass is observed only after puberty, and male CMs undergo greater hypertrophy than female CMs [11]. Men have 25–38% greater LV mass because of larger LV chamber dimension and wall thickness [11, 12]. In both sexes, the posterior wall thickness, septal wall thickness, LV mass, and LV mass index increase with age, but their values remain significantly smaller in women from 20 to 80 years of age [12]. The level of CM apoptosis also influences sex-specific differences; a male heart undergoes a higher percentage of apoptosis of ventricular CMs [13]. Thus, aging is associated with a significant decrease in the absolute number of CMs in a male heart, but the cellular volume is increased (i.e., hypertrophy) [14, 15]. In the left and right ventricles of males, binucleated cells progressively increase in number, whereas mononucleated cells progressively reduce with age [15]. Such phenomena have not been observed in a women heart [15]. The reason for the number of CMs to remain similar in an aging women heart but because of the higher regenerative capacity of the CMs in a women heart but is unlikely because of the absence of CM apoptosis. Females’ hearts are generally smaller but in proportion to their smaller body size [16].

### Cardiac functions 

LV ejection fraction (LVEF) is one of the central measures of LV systolic function. LVEF is the fraction of chamber volume ejected in systole (stroke volume) in relation to ventricular blood volume at the end of diastole (end-diastolic volume). Reports on whether LVEF differs between men and women are still conflicting [17].

The Framingham Heart Offspring Study showed that LVEF is not different between men and women [18], whereas the Dallas Heart Study reported that women had larger LVEF than men with the same age range [17]. Another large population study demonstrated that LVEF increased with age in both sexes, but was generally larger in women [12, 19]. After indexing ventricular volumes to body size, women have larger LVEF than men with similar age [20]. LV response to exercise also differs between sexes. Although the LVEF is smaller in men than in women at rest, it is reportedly larger in men than in age-matched women during exercise [21, 22]. Conversely, the increase in the end-diastolic volume index is greater in women during exercise but smaller during rest [22]. Additionally, men have a greater exercise capacity than women, most likely because they have a larger LV volume [23].

### Electrocardiogram and patterns

**Fig. 1:**
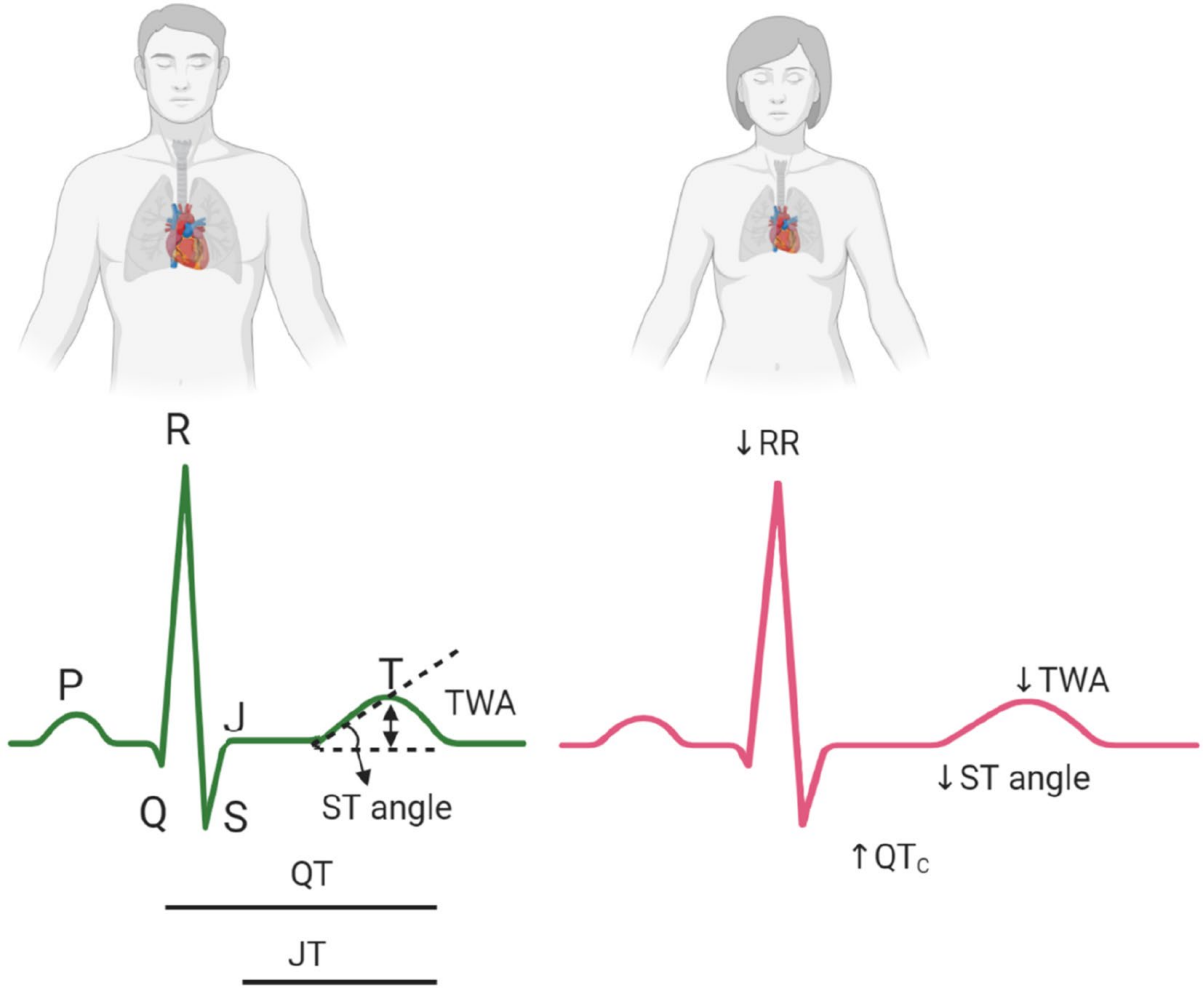
Sex differences in electrocardiogram (ECG). Interval between two consecutive Rs (RR), interval between Q and T wave (QT); interval between J and T wave (JT), rate-corrected QT interval (QT_C_,); angle of ST elevation (ST angle) and T-wave amplitude (TWA). Green and red ECGs from men and women, respectively

Electrocardiogram (ECG) is the most widely used clinical test to evaluate heart’s electrical activity. Atrial depolarization is seen as the P wave; subsequently, the right and left ventricles are depolarized, forming the QRS complex. The T-wave represents ventricular repolarization to the resting state. The normal QT interval depends on the heart rate, and it is typically corrected (QT_C_) using various correction formulas [24]. T-wave amplitude (TWA) is the highest peak of the T-wave. ST angle is the angle between the baseline and the first 80 ms of the ST segment. Similar to the QT interval, the JT interval is used to quantify the repolarization duration; it is defined as the time period of QT subtracted by QRS duration [25] (Fig. 1). In addition, QT dispersion is defined as the difference between the longest and shortest QT interval in different ECG leads.

Fetal heart rate examination at 20–36 weeks of age by echocardiography has revealed no sex-related differences [26]. Neonatal ECG has demonstrated that female babies have higher heart rates and shorter absolute values of the QT interval with a shorter cycle length, leading to a QT_C_ similar to that in male babies [27]. Studies with boys and girls younger than 15 years of age have shown no differences in heart rate and QT_C_ intervals [28, 29]. After puberty, the QT_C_ interval drops by approximately 20 ms in men, whereas it remains unchanged in women, leading to a longer QT_C_ in women irrespective of the correction methods [24, 29]. The QT_C_ gradually increases in men, and the correlation between age and QT_C_ is greater in men than in women [24]. Therefore, the QT_C_ differences between sexes are greater at younger ages, diminish with age, and disappear after 75 years of age [24, 28]. Furthermore, aging modulates the dispersion of ventricular repolarization; the older the individuals, the higher the QT_C_ dispersion, which may contribute to cardiac mortality in old population [30]. Although QT_C_ dispersion in young and middle-age shows no sex differences, it has been reported to be higher in older men than in older women (≥ 70 years old) [30, 31]. Sex differences are observed not only in cardiac repolarization, but also in ECG patterns (Fig. 1). Typical male and female patterns are readily recognizable in most ECGs without measurements. Male ECGs exhibit a higher J point, i.e., the level at which repolarization starts, a steeper ST segment (angle of the ST segment), and a greater TWA than female ECGs [25, 28]. Greater levels of J point and steepness of the ST angle segment in men implies a faster velocity of initial repolarization and typical early depolarization pattern [25]. During the period in which QT_C_ is longer in women, the distribution of men and women ECG patterns also shows differences [25, 28]. All these repolarization variables correlate negatively with age in men but not in women [25]. However, increasing age is associated with a higher heart rate and a shorter mean RR interval only in women [31–33]; this association is caused by differences in ambient autonomic tone [23]. In addition, the slopes of the linear relationship between the absolute values of QT and RR intervals are steeper in women, indicating that women have longer QT intervals than men at a decreased heart rate and that sex difference in QT_C_ is more marked at a longer cycle [33]. However, sex difference in QT_C_ is absent at a shorter cycle [33], possibly because women shorten their QT interval more than men in response heart rate increase [34].

In conclusion, sex differences in ECG and its patterns are apparent only after puberty. Men exhibit a shorter and faster repolarization after puberty until approximately 75 years of age. In both sexes, QT_C_ prolongs and ECG pattern changes with age, but these changes are more prominent in men than in women. Older men (> 70 years old) have a higher risk for ventricular arrhythmia at least partially due to higher QT_C_ dispersion.

### Effect of sex hormones and menstrual cycle 

Generally, women have significantly longer QT_C_ intervals because men begin to shorten their QT_C_ interval after puberty [29]. The different testosterone levels may explain the differences in QT interval between sexes, since testosterone accelerates ventricular repolarization [35, 36]. In addition, free testosterone at physiological levels inversely correlate with QT interval in men but not in women [35, 36]. Castrated males had longer rate-corrected JT intervals (JT_C_) and less steep ST-segments than non-castrated males, further supporting the role of testosterone on the configuration and duration of ventricular repolarization [37]. Furthermore, the age-dependent changes in male ECG pattern tend to correlate with the rise in testosterone level during puberty and the decline in older males [28].

Unlike testosterone, estrogen does not have clear effects on the duration and pattern of cardiac repolarization in humans [36]. Bilateral oophorectomy does not induce any ECG changes in postmenopausal females, but it increases the mean duration and decreases the amplitude of the T-wave in premenopausal women [38]. An estrogen does not alter the heart rate, QT_C_ or JT_C_ in resting condition, but estrogen-deficient state increases the QT interval dispersion, which decreases after hormonal replacement therapy suggesting the protective role of estrogen against severe ventricular arrhythmias and sudden cardiac death [39–41]. However, hormone replacement therapy with estrogen increases the QT interval dispersion during peak exercise [41]. When combined with progesterone, the QT_C_ interval does not increase, suggesting that progesterone reverses estrogen-induced QT prolongation [42]. Additionally, females with long QT syndrome (LQTS) have a lower risk for cardiac events during pregnancy, when the progesterone level increases [43]. However, the risk suddenly increases at postpartum, when the progesterone level decreases, also suggesting the protective role of progesterone [43]. In the menstrual cycle, the estrogen and progesterone levels change; estrogen levels gradually increase during the follicular phase, reach a peak after 11–13 days, and then decrease during the luteal phase, while progesterone levels do not increase until the luteal phase. Results about the effect of these hormonal changes on ECG and heart rate are still inconsistent [44–47].

In short, sex hormones, especially testosterone play an important role for the hormonal factor of the sex-specific differences mainly by affecting cardiac repolarization.

## Cellular level

Several studies have revealed that sex differences exist already at the cellular level. However, due to limited availability of human samples, most of the experiments have been conducted with animal models such as rodent and canine models. With the help of hiPSC technology, information about sex-specific differences in humans at the CMs level can be obtained.

### Contractility and excitation–contraction coupling

Cyclic changes in intracellular calcium (Ca^2+^) concentration regulate cardiac contractility. The Ca^2+^ influx through the L-type Ca^2+^ channels triggers the opening of ryanodine receptors (RyR2s), and subsequent release of Ca^2+^ from the sarcoplasmic reticulum (SR). This phenomenon gives rise to Ca^2+^ transients, referring to the process called Ca^2+^-induced Ca^2+^ release [48]. When the intracellular Ca^2+^ level increases, Ca^2+^ binds to myofilaments (troponin C), causing CM contraction. Relaxation occurs when the majority of Ca^2+^ is transferred back into the SR via the SR Ca^2+^-ATPase (SERCA), while a smaller amount of Ca^2+^ is extruded from the cell predominantly by the sodium (Na^+^)/Ca^2+^ exchanger (NCX) [48].

At the cellular level, sex differences are observed in excitation–contraction coupling (ECC). Table 1 compares the intracellular Ca^2+^ handling properties between female and male CMs of different species. Several experimental results have shown that the CMs from female heart samples exhibit a smaller contraction along with longer time both to maximal/peak shortening and to 50% relaxation [49–53]. CM contractility depends not only on the myofilaments properties, but also on the diastolic and systolic Ca^2+^levels. The smaller contraction in female CMs arises from the smaller Ca^2+^ transient amplitude and/or lower diastolic Ca^2+^ levels in female CMs [49–54]. Another characteristic of female CMs is the slower rate of rise and decay of Ca^2+^ transient, corresponding to a longer time to peak shortening and the slower time to 50% relaxation [49, 50].

**Table 1:** Intracellular Ca^2+^ handling properties in female cardiomyocytes compared to male cardiomyocytes

Parameters	Differences	Species	Source	Age	[Ca^2+^] (mM)	Ca^2+^ dye	Temp.	Refs.
Ca^2+^ transient amplitude	**↓**	Rat	V	~ 50 days	1.8	Fura-2 AM	37 °C	[50]
	**↓**	Mice	V	5–10 months	1	Fura-2 AM	37 °C	[54]
	**↓**	Rat	LV	~ 3 months	0.5,1,1.5 and 2	Fura-2 AM	25 °C	[49]
	** ↔ **	Mice	V	~ 7 and 24 months	1	Fura-2 AM	37 °C	[51]
	** ↔ **	Rat	V	~ 3 months	1.8	Fura-2 AM	37 °C	[52]
Ca^2+^ transient time-to-peak	**↑**	Rat	LV	~ 3 months	0.5,	Fura-2 AM	25 °C	[49]
	**↑**	Rat	V	~ 50 days	1.8	Fura-2 AM	37 °C	[50]
	**↑**	Rat	V	~ 3 months	1.8	Fura-2 AM	37 °C	[52]
	** ↔ **	–	–	~ 24 months	–	–	–	–
Ca^2+^ transient rise rate	**↓**	Rat	V	~ 50 days	1.8	Fura-2 AM	37 °C	[50]
	**↓**	Rat	V	~ 3 months	1.8	Fura-2 AM	37 °C	[52]
	** ↔ **	–		~ 24 months	–	–	–	–
	** ↔ **	Mice	V	~ 7 and 24 months	1.5	Fura-2 AM	37 °C	[51]
Ca^2+^ transient 90% decay time	**↓**	Rat	V	~ 24 months	1.8	Fura-2 AM	37 °C	[52]
	** ↔ **	–		~ 3 months	–	–	–	–
	** ↔ **	Rat	V	~ 50 days	1.8	Fura-2 AM	37 °C	[50]
Ca^2+^ transient decay rate	**↓**	Rat	LV	~ 3 months	1.5	Fura-2 AM	25 °C	[49]
Diastolic Ca^2+^	**↓**	Rat	V	~ 50 days	1.8	Fura-2 AM	37 °C	[50]
	**↓**	Rat	V	~ 3 months	1.8	Fura-2 AM	37 °C	[52]
	** ↔ **	–		~ 24 months	–	–	–	–
	** ↔ **	Mice	V	5–10 months	1	Fura-2 AM	37 °C	[54]
	** ↔ **	Mice	V	~ 7 months	1	Fura-2 AM	37 °C	[51]
	**↑**			~ 24 months				
Sarcoplasmic reticulum Ca^2+^ content	** ↔ **	Rat	V	~ 50 days	1.8	Fura-2 AM	37 °C	[50]
	** ↔ **	Mice	V	5–10 months	1	Fura-2 AM	37 °C	[54]
	**↑**	Guinea pig		7 weeks	1	NA	37 °C	[55]
E-C coupling gain	**↓**	Rat	V	~ 50 days	1.8	Fura-2 AM	37 °C	[50]
	**↓**	Mice	V	5–10 months	1	Fura-2 AM	37 °C	[54]
	**↓**	Rat	V	~ 3 months	1.8	Fura-2 AM	37 °C	[52]
	** ↔ **	–	–	~ 24 months	–	–	–	–
	** ↔ **	Mice	V	~ 7 and 24 months	1	Fura-2 AM	37 °C	[51]
Ca^2+^ Spark frequency	** ↔ **	Rat	V	~ 50 days	1.8	Fluo-4 AM	37 °C	[50]
	** ↔ **	Mice	V	5–10 months	1	Fluo-4 AM	37 °C	[54]
Ca^2+^ Spark amplitude	**↓**	Rat	V	~ 50 days	1.8	Fluo-4 AM	37 °C	[50]
	**↓**	Mice	V	5–10 months	1	Fluo-4 AM	37 °C	[54]
Ca^2+^ Spark decay	**↓**	Rat	V	~ 50 days	1.8	Fluo-4 AM	37 °C	[50]
	**↓**	Mice	V	5–10 months	1	Fluo-4 AM	37 °C	[54]
Ca^2+^ Spark duration	**↓**	Rat	V	~ 50 days	1.8	Fluo-4 AM	37 °C	[50]
	** ↔ **	Mice	V	5–10 months	1	Fluo-4 AM	37 °C	[54]

*V* ventricle, *LV* left ventricle

Smaller and slower rise of Ca^2+^ transient in female CMs implies reduced RyR2 activity, while a slower decay is due to reduced SERCA and/or NCX activity. Ca^2+^ content in SR is the same in male and female CMs and thus this does not explain the smaller Ca^2+^ release in female CMs [50, 54]. Actually, SR Ca^2+^ content has even been found to be higher in female CMs in one study [55]. The protein levels of SERCA, phospholamban (PLB), and calsequestrin show no sex differences [56, 57]. However, the protein and mRNA levels of RyR2 and NCX are higher in female hearts [57]. Thus, the altered intracellular Ca^2+^ handling in female CMs is not caused by the SR Ca^2+^ content and Ca^2+^ handling protein levels. Individual SR Ca^2+^ release units known as Ca^2+^ sparks may partly explain for this disparity. Female CMs have smaller Ca^2+^ spark amplitudes and durations (time to peak and decay time) [50, 54]. Furthermore, the degree of amplification of Ca^2+^ influx to the resulting amount of Ca^2+^ released from the SR can be quantified as ECC gain, which indicates the ratio of the amount of SR Ca^2+^ released per unit of L-type Ca^2+^ current (*I*_CaL_). In female CMs, the EEC gain is weaker, demonstrating that the amount of Ca^2+^ release per unit *I*_CaL_ is lower in female CMs [53, 55]. Therefore, the smaller Ca^2+^ transient amplitude in female CMs is more likely to be caused by reduced Ca^2+^ influx trigger with a consequent reduction in SR Ca^2+^ release [49]. Aging also affects the sex differences in ECC [51, 52]. The fractional shortening, *I*_CaL_, and Ca^2+^ transients are reduced only in the CMs of older male mice, whereas SR Ca^2+^ content is increased in the CMs of older female mice [51]. Moreover, the Ca^2+^ sensitivity of myofilaments declines with age in male CMs, causing smaller contractions in older male CMs [52]. Therefore, despite that younger female CMs demonstrate smaller contractions, Ca^2+^ transient amplitude, diastolic Ca^2+^, and ECC gain, such differences vanish as the age advances [51, 52]. In addition, intracellular signaling and/or sex hormones play an important role in the regulation of intracellular Ca^2+^ handling, which engender sex-specific differences, as described below.

Taken together, the ECC in CMs depends on both sex and age, and the age-related alterations are more prominent in males than in females. Of note, smaller Ca^2+^ sparks and lower ECC gain are fundamental characteristics of female CMs. The marked reduction in ECC gain in female CMs may limit SR Ca^2+^ release under physiological conditions of stress such as exercise; this may explain why females are less capable of increasing LVEF under stress or exercise than males.

### Action potential and ionic currents 

Sex differences have been observed in action potential (AP) parameters and ionic currents and are strongly dependent on the species and origin of CMs within the ventricular wall (Table 2). In some animal studies, AP duration (APD) is longer in female CMs than in male CMs [55, 58–61], but other studies found no sex difference in APD [50, 53, 62, 63]. Xiao and coworkers examined sex differences at three transmural levels in dogs and demonstrated that APD was only significantly longer in the mid-myocardium of female dogs; the epicardium and endocardium showed no sex differences [64]. The resting membrane potential (RMP) [50, 53, 58, 60, 61, 63, 64], maximal upstroke velocity (dV/dt) [63], and AP amplitude (APA) [55, 63] are sex independent. Fast Na^+^ current (*I*_Na_) is responsible for the rapid upstroke phase of AP. Although the *I*_Na_ densities in dog mid-myocardium do not differ in sexes [65], those in female endo/epicardium were significantly lower than those in male endo/epicardium [65].

**Table 2:** Action potential parameters and ionic currents in female cardiomyocytes compared to male cardiomyocytes

Parameters	Differences	Species	Origin	Age	Temperature	References
APD	**↑**	Human	LV-Mid	17–60 years	36 ± 0.5 °C	[71]
	**↑**	Guinea pig	LV	7 weeks	37 °C	[55]
	**↑**	Guinea pig	V	NA	35 °C	[59]
	**↑**	Dog	LV-Mid	NA	36 °C	[64]
	** ↔ **		LV-Epi/Endo			
	**↑**	Rabbit	LV	~ 6 months	RT	[60]
	**↑**	Mouse	LV-, RV-Epi	2–3 months	RT	[61]
	**↑**	Mouse	LV	10–12 months	35 ± 1 °C	[58]
	** ↔ **	Guinea pig	LV	13–17 weeks	36 ± 1 °C	[62]
	** ↔ **	Rabbit	RV-Epi	50–60 days	37 °C	[63]
	** ↔ **	Rat	V	~ 50 days	37 °C	[50]
	** ↔ **	Rat	LV	3 and 9 months	35 °C	[53]
Cell length	**↓**	Rat	V	NA	37 °C	[74]
	**↓**	Rat	V	~ 3 and ~ 24 months	37 °C	[52]
	** ↔ **	Rat	V	~ 50 days	37 °C	[50]
	** ↔ **	Mouse	V	~ 7 and ~ 24 months	37 °C	[51]
C_m_	** ↔ **	Human	LV-Mid	17–60 years	36 ± 0.5 °C	[71]
	** ↔ **	Rat	V	~ 50 days	37 °C	[50]
	** ↔ **	Mouse	V	5–10 months	37 °C	[54]
	** ↔ **	Guinea pig	LV	7 weeks	37 °C	[55]
	** ↔ **	Rat	V	NA	37 °C	[74]
	** ↔ **	Rat	V	~ 3 and ~ 24 months	37 °C	[52]
	** ↔ **	Mouse	LV-, RV-Epi	2–3 months	RT	[61]
	** ↔ **	Dog	LV-Epi/Mid/Endo	NA	36 °C	[64]
	** ↔ **	Mouse	V	~ 7 and ~ 24 months	37 °C	[51]
	**↓**	Rat	LV	3,6 and 9 months	35 °C	[53]
	**↓**	Mouse	LV	10–12 months	35 ± 1 °C	[58]
APA	** ↔ **	Human	LV-Mid	17–60 years	36 ± 0.5 °C	[71]
	** ↔ **	Rabbit	RV-Epi	50–60 days	37 °C	[63]
	** ↔ **	Guinea pig	LV	7 weeks	37 °C	[55]
d*V*/d*t*	** ↔ **	Human	LV-Mid	17–60 years	36 ± 0.5 °C	[71]
	** ↔ **	Rabbit	RV-Epi	50–60 days	37 °C	[63]
RMP	** ↔ **	Human	LV-Mid	17–60 years	36 ± 0.5 °C	[71]
	** ↔ **	Rat	V	~ 50 days	37 °C	[50]
	** ↔ **	Rabbit	LV	~ 6 months	RT	[60]
	** ↔ **	Mouse	LV-, RV-Epi	2–3 months	RT	[61]
	** ↔ **	Dog	LV-Epi/Mid/Endo	NA	36 °C	[64]
	** ↔ **	Rat	LV	3 and 9 months	35 °C	[53]
	** ↔ **	Mouse	LV	10–12 months	35 ± 1 °C	[58]
	** ↔ **	Rabbit	RV-Epi	50–60 days	37 °C	[63]
*I*_Na_	** ↔ **	Mouse	LV-, RV-Epi	2–3 months	RT	[61]
	** ↔ **	Dog	LV-Mid	NA	RT	[65]
	**↓**	Dog	LV-Endo/Epi	–	–	–
*I*_to_	**↓**	Mouse	LV	10–12 months	35 ± 1 °C	[58]
	**↓**	Dog	LV-Endo	NA	36 °C	[64]
	** ↔ **	–	LV-Epi/Mid	–	–	–
	** ↔ **	Mouse	LV-, RV-Epi	2–3 months	RT	[61]
*I*_CaL_	**↑**	Human	LV-Mid	17–60 years	36 ± 0.5 °C	[71]
	**↑**	Guinea pig	LV	7 weeks	37 °C	[55]
	**↑**	Dog	LV-Epi/Mid/Endo	NA	36 °C	[64]
	**↓**	Guinea pig	V	NA	35 °C	[59]
	** ↔ **	Mice	V	5–10 months	37 °C	[54]
	** ↔ **	Rat	V	~ 50 days	37 °C	[50]
	** ↔ **	Mouse	LV-, RV-Epi	2–3 months	RT	[61]
	** ↔ **	Rat	LV	3 and 9 months	35 °C	[53]
	** ↔ **	Guinea pig	LV	13–17 weeks	36 ± 1 °C	[62]
	** ↔ **	Rat	V	NA	37 °C	[74]
*I*_Ks_	**↓**	Rabbit	LV	~ 6 months	RT	[60]
	**↑**	Dog	LV-Epi/Endo	NA	36 °C	[64]
	** ↔ **	–	LV-Mid	–	–	–
	** ↔ **	Guinea pig	LV	13–17 weeks	36 ± 1 °C	[62]
	** ↔ **	Guinea pig	LV	7 weeks	37 °C	[55]
*I*_Kr_	** ↔ **	Guinea pig	LV	7 weeks	37 °C	[55]
	**↓**	Rabbit	V	3–4 months	RT	[67]
	** ↔ **	Dog	LV-Epi/Mid/Endo	NA	36 °C	[64]
	** ↔ **	Guinea pig	LV	13–17 weeks	36 ± 1 °C	[62]
*I*_K1_	**↓**	Guinea pig	V	NA	35 °C	[59]
	**↓**	Rabbit	V	3–4 months	RT	[67]
	** ↔ **	Dog	LV-Epi/Mid/Endo	NA	36 °C	[64]
	** ↔ **	Guinea pig	LV	13–17 weeks	36 ± 1 °C	[62]
	** ↔ **	Guinea pig	LV	7 weeks	37 °C	[55]
	** ↔ **	Mouse	LV-, RV-Epi	2–3 months	RT	[61]
*I*_Kur_	**↓**	Mouse	LV-, RV-Epi	2–3 months	RT	[61]

*APD* action potential duration, *C*_*m*_ cell capacitance, *APA* action potential amplitude, *dV/dt* maximal upstroke velocity, *RMP* resting membrane potential, *I*_*Na*_ sodium current, *I*_*to*_ transient outward potassium current, *I*_*CaL*_ L-type calcium current, *I*_*Ks*_ slowly delayed rectifier potassium current, *I*_*Kr*_ rapidly delayed rectifier potassium current, *I*_*K1*_ inward rectifier potassium current, *I*_*Kur*_ ultra-rapid delayed rectifier potassium current, *V* ventricle, *LV* left ventricle, *RV* right ventricle, *Epi* epicardium, *Mid* midcardium, *Endo* endocardium, *RT* room temperature

Following rapid depolarization, transient outward potassium (K^+^) current (*I*_to_) starts the repolarization of AP. The *I*_to_ densities are significantly lower in female mouse CMs [58] and in female dog endocardium than in male counterparts [64]. The *I*_CaL_ is responsible for the plateau phase of AP. Female CMs from dogs [64] and guinea pigs [55] have increased *I*_CaL_ densities. In contrast, James et al. showed that *I*_CaL_ densities are decreased in female guinea pig CMs [59]. Furthermore, Sims and coworkers demonstrated significantly larger *I*_CaL_ densities in CMs from the base of adult female rabbit hearts but found no difference between apical CMs [66]. As the plateau phase moves toward a more negative membrane potential, two types of K^+^ currents, namely, rapid rectifier K^+^ current (*I*_Kr_) and slow rectifier K^+^ current (*I*_Ks_), are activated. According to Liu et al., female rabbit CMs have significantly lower *I*_Kr_ densities than male rabbit CMs [67]. Zhu et al. also showed significantly lower *I*_Ks_ densities in female rabbit CMs [60]. Conversely, Xiao et al. demonstrated higher *I*_Ks_ densities in female dog epicardium and endocardium [64]. The ultra-rapid delayed rectifier K^+^ current (*I*_Kur_) is mainly responsible for repolarization in mouse CMs, and *I*_Kur_ densities are significantly lower in female mouse CMs than in male CMs [61]. The inward rectifier K^+^ current (*I*_K1_) is activated during and after the repolarization phase to ensure terminal repolarization and stable RMP. The *I*_K1_ densities are significantly smaller in female guinea pig [59] and rabbit CMs [67]. Moreover, NCX current (*I*_NCX_) densities are similar in sexes in pig [68] and rabbit atrial CMs [69] and CMs from the apex of a rabbit heart [70], but *I*_NCX_ densities are higher in female CMs from the base of a rabbit heart [70]. Verkerk et al. obtained human CMs from failing hearts and showed that female CMs have significantly longer APD and greater susceptibility to early after depolarization (EAD) [71]. In addition, female CMs express higher *I*_CaL_ densities but smaller *I*_to_ densities than male CMs, but the APA, dV/dt, and RMP show no sex differences [71]. Gaborit et al. obtained CMs from healthy human hearts and demonstrated that female CMs express lower levels of various genes (human ether-a-go-go-related gene, mink, Kir2.3, Kv1.4, KChIP2, SUR2, and Kir6) responsible for cardiac repolarization [72].

Overall, many animal and human experiments have shown that APDs from female CMs are longer than that from male CMs, consistent with the clinical observation that women have longer QTc intervals. The cardiac AP results from the complex interplay of time- and voltage-dependent inward and outward ionic currents during AP’s various phases. The difference in APD between male and female CMs results from the unique composition of the ionic currents governing the APD. Furthermore, female CMs are more susceptible to EADs in response to increased cycle length, supporting the clinical observation that female sex is an independent risk factor for TdP [71].

### Effect of beta-adrenergic stimulation 

Adrenergic receptor activation is the primary mechanism that increases cardiac performances under stress. Upon beta-adrenergic activation, adrenergic receptors couple with G proteins, leading to adenylyl cyclase activation and secondary-messenger cyclic adenosine monophosphate (cAMP) production. Subsequently, cAMP activates protein kinase A (PKA), which promotes phosphorylation in various substrates, such as (i) L-type Ca^2+^ channel, which increases Ca^2+^ entry into cardiomyocytes; (ii) PLB, which accelerates Ca^2+^ sequestration into the SR and cardiac relaxation, and (iii) troponin I and C proteins, which reduce myofilament sensitivity to Ca^2+^ [73].

Male CMs exhibit higher beta-adrenergic receptor density and basal intracellular cAMP levels than female CMs; therefore, they have larger cAMP production upon beta-adrenergic stimulation [54, 74]. Furthermore, beta-adrenergic stimulation causes a larger augmentation of *I*_CaL_ current densities, leading to increased Ca^2+^ release from SR, although basal SR Ca^2+^ content is similar between male and female CMs [49, 54, 56, 74]. The enhanced response to beta-adrenergic stimulation due to augmented intracellular signaling explains the larger positive inotropic effect in male CMs, as observed in both ventricular [74] and atrial CMs [75]. Additionally, the reduction in the decay time constant of the Ca^2+^ transient caused by beta-adrenergic stimulation is higher in the male heart than in the female heart, revealing that male hearts have a more pronounced lusitropic effect (i.e., relaxation) [76]. Even though the male and female hearts have similar Ca^2+^ transient durations after beta-adrenergic stimulation, APD reduction is less prominent in the female heart, as shown in the simultaneous optical mapping in ventricular AP and Ca^2+^ transient recording [76]. Voltage-clamp study revealed that beta-adrenergic stimulation induces smaller *I*_Ks_ in female CMs, suggesting the underlying mechanism behind the reduced capability of female CMs to further decrease in APD upon beta-adrenergic stimulation [60].

In conclusion, lower beta-adrenergic receptor density and/or lower intracellular cAMP level in female CMs attenuates the PKA phosphorylation of Ca^2+^ handling proteins and ion channels, which is the putative mechanism behind the limited positive inotropic effect under beta-adrenergic stimulation. The preserved beta-adrenergic regulation might be associated with reduced arrhythmic activity, explaining why women are less prone to severe arrhythmias [76].

## Influence of sex hormones

**Fig. 2:**
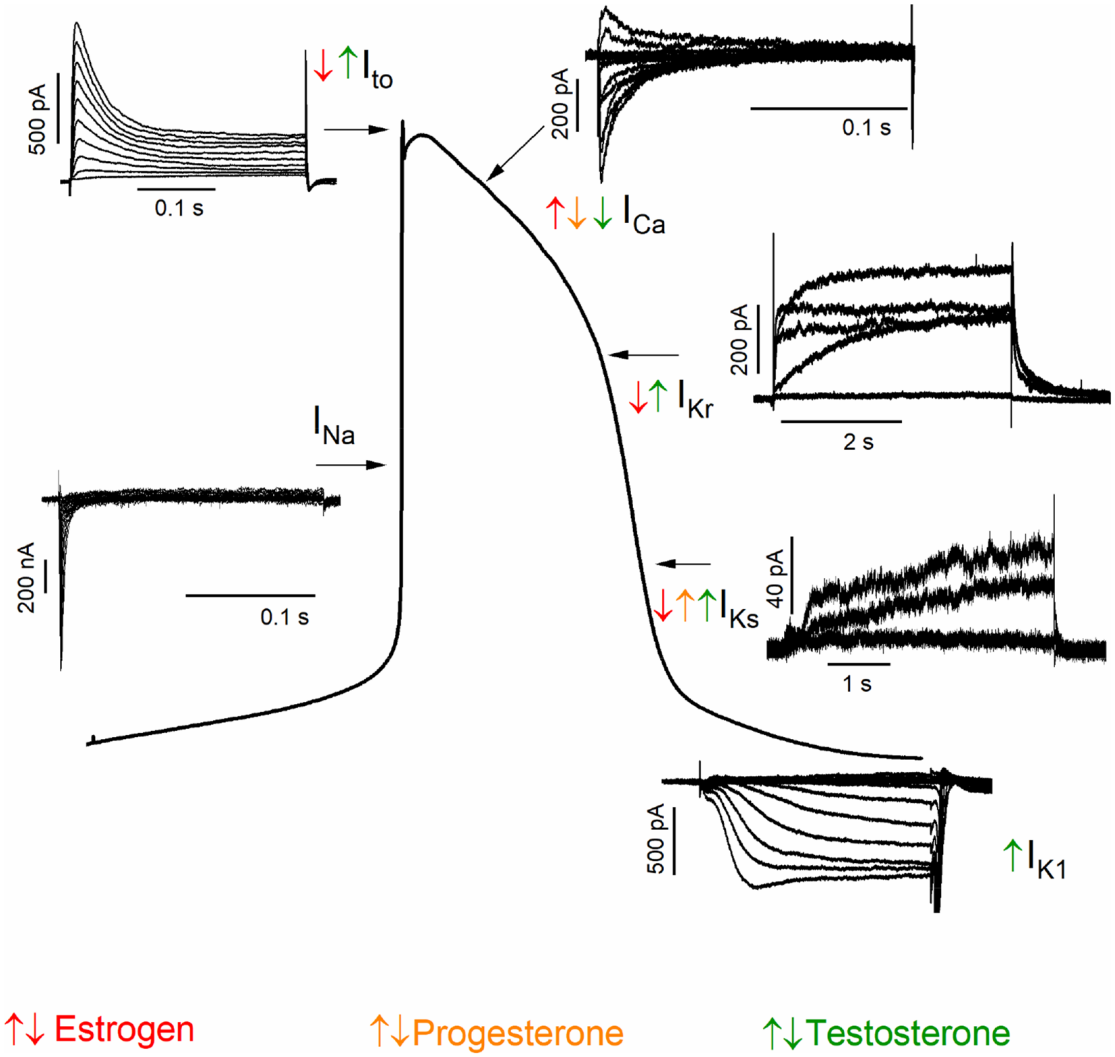
Different voltage-gated ionic currents involved in action potential and influence of sex hormones in these ionic current. *I*_to_, transient outward potassium current; *I*_Ca_, calcium current; *I*_Na_, sodium current; *I*_Kr_/*I*_Ks_, rapid/slow rectifier potassium current; *I*_K1_, inward rectifier potassium current

The effect of sex hormones on cardiovascular physiology has been widely studied. CMs express sex hormone receptors, indicating that these hormones have direct cardiac effects [77–79]. At cellular level, sex hormones regulate various voltage-gated ion channels (Fig. 2) and also intracellular Ca^2+^ handling, thereby altering the cardiac repolarization [80].

### Effect of sex hormones on ion channels

**Fig. 3:**
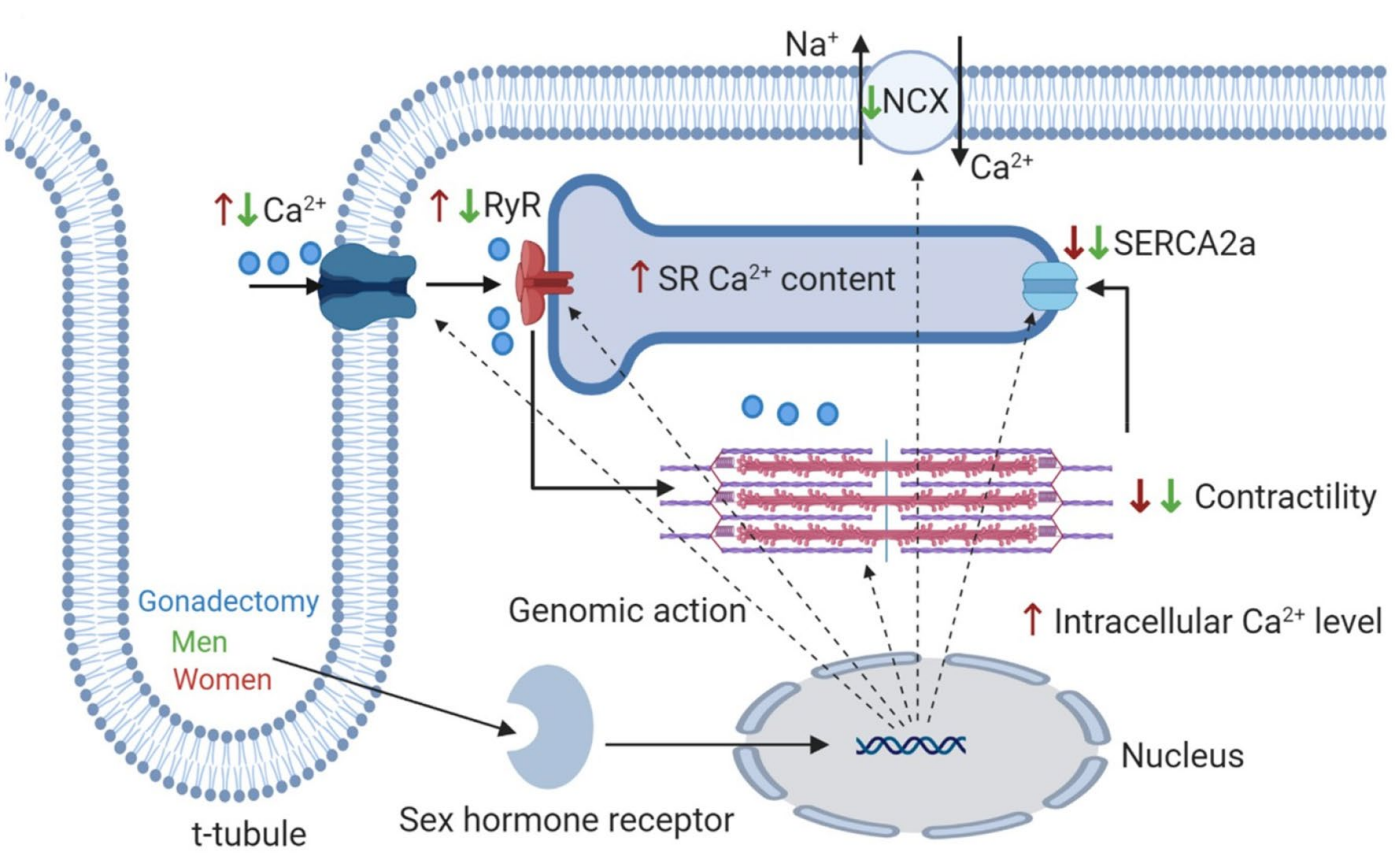
Effect of gonadectomy in excitation–contraction coupling. Red arrows represent the effect of gonadectomy in women and green color represents the effect of gonadectomy in men

The effects of sex hormones on ion channels depend mainly on whether the hormone is present acutely or chronically (Fig. 3). The acute exposure of testosterone within the physiological range rapidly reduces the open probability of single *I*_CaL_ and lowers *I*_CaL_ current density [81]. It also decreases the APD mainly by enhancing *I*_Ks_ and suppressing *I*_CaL_ via nitric oxide synthase 3 activation and nitric oxide production through a nongenomic pathway [82]. In contrast, the chronic treatment of testosterone increases the single-channel activity of *I*_CaL_ and *I*_CaL_ current density [81] and also *I*_Na_ current density [65]. Furthermore, testosterone upregulates the expression of genes for *I*_CaL_ and NCX [83]. In female guinea pig CMs, the acute application of progesterone at 100 nM/L reduces APD mainly by enhancing *I*_Ks_ and inhibiting *I*_CaL_ via a nongenomic pathway [84]. However, the supraphysiological concentration of progesterone (1–30 μM) in Langendorff-perfused female rabbit hearts exhibits a biphasic effect; it prolongs monophasic APD at lower concentrations (1–3 μM) but shortens at higher concentrations (10–30 μM) [85]. Moreover, high estradiol concentrations (1–30 μM) prolong the APD in a concentration-dependent manner by affecting one or more of the ionic currents, including *I*_CaL_, *I*_Kr_, *I*_Ks_, *I*_to_, and *I*_K1_ [85, 86]. Estradiol can directly interact with ion channels without involving the membrane-associated estrogen receptors [87]. Of note, high estradiol concentrations prolong the APD by inhibiting *I*_Kr_ and *I*_Ks_ in female guinea pig CMs [88], but shorten it by inhibiting *I*_CaL_ and delaying the recovery time of *I*_CaL_ in male guinea pig CMs [89]. Therefore, while studying the effect of hormones on CMs, the sex of the animal from where CMs are isolated, should be considered. The estrogen of 300 nM concentrations shortens the APD by enhancing *I*_Ks_ and suppressing *I*_CaL_ [90], but its physiological concentration (1 nM) prolongs the APD by suppressing *I*_Kr_, with little or no effect on *I*_Ks_ and *I*_CaL_ [90]. Furthermore, the incubation of CMs with estradiol (1 nM) enhances NCX current [70]. The plasma estrogen concentration is one of the key players in determining outward K^+^ current density thus, it affects the ventricular repolarization, as confirmed by reduced total outward K^+^ current densities and downregulation of K^+^ channel transcript level in female CMs [91, 92]. The long-term deficiency of ovarian hormones after ovariectomy results in higher *I*_CaL_, creating a larger “window” current that facilitates the increased occurrence of arrhythmias with and without isoprenaline in female guinea pig CMs [93]. However, in same study, the estradiol replacement prevents arrhythmias in CMs from ovariectomized guinea pig [93].

In conclusion, sex hormones directly regulate ion channels and alter APD depending on their concentrations and whether the exposures are short or long. The long-term exposure of sex hormones acts via a genomic pathway, in which sex hormones bind to sex hormone receptors, translocate into nucleus and lead to the transcriptional regulation of ion channels [81, 94]. In addition, sex hormones can also directly targets the ion channels in a receptor-independent manner and referred as nongenomic regulation [81, 94]. Nongenomic actions can be distinguished from genomic effects by a more rapid onset (seconds to minutes), take place outside the cell nucleus via the activation of intracellular signaling including endothelial nitric oxide synthase and mitogen-activated protein kinase, and the fact that observed effects may not be blocked by sex hormone receptor antagonists [81, 94].

### Effect of sex hormones on intracellular Ca^2+^ handling and contractility

The sex hormones also modulate the intracellular Ca^2+^ handling and contractile properties of CMs depending on acute or chronic application of hormones (Fig. 3). Pretreatment of rat CMs with testosterone (100 nM) for 24–30 h increases the peak Ca^2+^ transients, frequency of Ca^2+^ sparks, and fractional shortening thus it improves the CM contractility without altering the SR Ca^2+^ load [81]. In contrast, the acute testosterone application decreases the frequency of Ca^2+^ sparks and reduces contractility in testosterone-pretreated rat CMs [81]. When testosterone (100 nM) is acutely applied to non-testosterone-pretreated neonatal rat CMs, the intracellular Ca^2+^ release from the intracellular storage is increased by elevating the inositol 1,4,5-trisphosphate level [95]. In isolated male rat ventricular CMs, 24-h exposure of testosterone (1 μM) increases the peak shortening and relaxation velocity and decreases the time to peak shortening; conversely, acute exposure has no effect on the contraction and relaxation properties [79]. Animal experiments on gonadal testosterone withdrawal (GDX) have been conducted to study the influence of endogenous gonadal testosterone on the regulation of the Ca^2+^ handling in the heart. Two weeks after GDX reduced Ca^2+^ transient amplitude and peak shortening of CMs, and slowed down the Ca^2+^ transient decay is observed in male rats; however, the effects were completely reversed by testosterone replacement [96]. The reduction of the mRNA levels for the genes of NCX and L-type Ca^2+^ channels is observed [97]. However, long-term testosterone deficiency (10 weeks after GDX) increases the NCX1 expression, but it does not change the expression of SERCA2a, CSQ2, or total PLB in male mice [98]. If male rats receive testosterone replacement 9 weeks after GDX, the contractility and Ca^2+^ transient amplitude increase as a result of high Ca^2+^ release from SR and more efficient Ca^2+^ reuptake through SERCA and removal via NCX [99].

Moreover, acute estradiol application at supraphysiological concentrations (10–30 μM) reduces the contraction and Ca^2+^ transient amplitude [89]; the estrogen-induced negative inotropic effect is not mediated via estrogen receptors in the membrane, but it is more on the direct inhibition of *I*_CaL_ [87]. Ovariectomy in female animals results in higher Ca^2+^ transient amplitude, faster rise and decay of Ca^2+^ transient, higher SR Ca^2+^ content, greater ECC gain, and higher spark frequencies in isolated CMs [93, 100, 101]. Ovariectomy increases SR Ca^2+^ storage and promotes SR Ca^2+^ loading, thus resulting into the increased Ca^2+^ spark frequency in ovariectomized CMs [101]. Furthermore, CMs from an ovariectomized animal heart exhibit decreased peak shortening, reduced maximum shortening/relengthening velocity, and prolonged shortening/relengthening duration [102]. The slow intracellular Ca^2+^ clearing and elevated resting intracellular Ca^2+^ levels caused by SERCA/PLB protein downregulation may underlie the mechanism behind the estrogen deficiency-induced altered mechanical and intracellular Ca^2+^ homeostasis [102, 103]. The estradiol [93, 102, 104] or progesterone [103] replacement in ovariectomized female animals ameliorates the changes in contractile function and intracellular Ca^2+^ handling properties of CMs caused by ovariectomy. Estrogen deficiency promotes the intracellular Ca^2+^ dysregulation, reduces myofilament Ca^2+^ sensitivity, and alters the contractile function, causing the formation of a more proarrhythmic substrate in an aging female heart. Taken together, the sex hormones directly modulate the myocardial function which partially explain the sex-based differences in myocardial function and may help to determine the differential incidence and outcomes of cardiac disease conditions in men and women.

## Cardiovascular drug responses 

Women have a higher risk of developing a special type of ventricular tachycardia, TdP, associated with adverse effects of drugs that prolong ventricular repolarization time. This is due to several sex-specific differences in physiology, pharmacokinetics, and/or pharmacodynamics [105]. Typically, women not only have a longer QT_C_ at baseline, but also have more prolonged QT intervals after taking drugs known to prolong cardiac repolarization (e.g., Class I and III antiarrhythmic drugs), placing them at a higher risk for TdP [105]. However, the detailed mechanism of TdP remains poorly understood. One possible explanation could be that drug concentrations are higher in women because of their smaller body sizes. However, quinidine (Class Ia antiarrhythmic drug) causes greater QT_C_ prolongation in women than in men, although its serum concentrations and pharmacokinetics are not different between men and women [106, 107]. Furthermore, d,l-sotalol (class III antiarrhythmic drugs) increases the mean heart rate in women, who also face a threefold increased risk of developing TdP [108]. One study demonstrated that d,l-sotalol could induce extreme JT_C_ prolongation in women regardless of the baseline JT_C_ [109]. In another study investigating dofetilide (class III antiarrhythmic drug) administration and creatinine clearance in age-matched men and women, more than half of women discontinued the drug or underwent dose reduction because of significant QT_C_ prolongation [110]. The Digitalis Investigation Group evaluated the efficacy of digoxin therapy in patients with heart failure (HF) and found that women taking digoxin had a higher mortality rate, although women with HF, but without digoxin medication had a lower mortality risk than men [111].

The endogenous sex hormones also have a role in drug-induced QT prolongation. After ibutilide (class III antiarrhythmic drug) infusion, the QT_C_ prolongation and TdP occurrence was higher in women than in men [112, 113]. However, the progesterone level inversely correlates with the mean ibutilide-induced QT_C_ prolongation and ibutilide-induced QT_C_ prolongation is shortest during the luteal phase compared with that during the menstrual and ovulation phases, suggesting the possible protective effect of progesterone [113]. Furthermore, drug response in genetic cardiac diseases also differs with sex. During beta-blocker treatment of LQT type 1 patients with similar QT_C_ intervals, men have shorter QT_C_ intervals than women [114]. Moreover, beta-blocker therapy reduces the cardiac event rates in women with LQT type 3 syndrome, but its efficacy in men is still inconclusive because of the low number of prior cardiac events [115]. Patients with Na^+^ loss-of-function condition exhibit cardiac conduction disturbances and could provoke Brugada syndrome. Women with Brugada syndrome show greater conduction intervals and QT_C_ in response to Na^+^ channel blockers, even though their baseline ECG parameters are similar to those of men [116].

Several in vitro studies have been performed to understand sex-specific differences in drug responses, and the results are similar to those of in vivo studies. Animal studies showed that a female heart is more prone to TdP development subsequent to QT prolongation in response to E-4031 (*I*_Kr_ blocker) and 4-aminopyridine (*I*_to_ blocker) [66, 117]. Dofetilide (*I*_Kr_ blocker) administration induces greater APD prolongation, EAD incidence, and repolarization dispersion in CMs from female hearts [63]. In addition, CMs from a gonadectomized male heart have higher dofetilide-induced APD prolongation and the presence of EADs than those from a control male heart. Testosterone replacement in gonadectomized animals diminishes the effect of *I*_Kr_ blockers on QT and APD, whereas estrogen replacement increases blockers’ adverse effects such as a higher magnitude of drug-induced prolongation and EAD incidences [63, 92, 118].

In conclusion, women have larger drug-induced QT_C_ prolongation than men, and sex hormones play an important role in cardiac repolarization. All these results suggest that female sex is an independent risk factor for increased cardiac side effects of various drugs, and women have especially an increased risk of developing TdP. Therefore, the QT interval in female patients receiving drugs with potential effects on cardiac repolarization should be monitored closely.

## Potential of human stem cell-based approaches

Considering the limited human heart material for research, most of our knowledge about sex-specific differences relies on animal experiments. However, cardiac ion channels, Ca^2+^ handling components, and estrous cycle differ among species. Although animal experiments reveal interesting results, direct extrapolation of animal data to humans is not always possible because of several uncertainties. Insufficient human-based data extensively limit our understanding of sex-specific electrophysiology, and further studies with human-based experiments are urgently required. The recent methodological and technical advancements with iPSC technology offer a robust and human-based platform for studying human cardiomyocyte physiology and pathophysiology [119, 120] as well as for screening pharmacological compounds [121]. HiPSCs provide an unlimited source of human CMs, offering an accessible and robust platform to study sex-specific drug responses. One of their advantages is that they carry donor-specific genetic information, enabling researchers to study the mechanisms of sex differences in different genetic cardiac diseases. One limitation of currently used hiPSC-CMs is that their phenotype resembles embryonic CMs [122] warranting caution in interpretation of the results. However, hiPSC-CMs have been successfully used to investigate the influence of genetics and sex hormones on the gene expression of cardiac ion channels and function of CMs. Papp and coworkers were the first to study the effect of sex hormones in hiPSC-CMs; they found that estradiol increases the *I*_CaL_ and NCX current densities in hiPSC-CMs obtained from a female donor, and it slightly increases the *I*_CaL_ in male-origin hiPSC-CMs but does not affect current NCX densities [123]. In clinical studies, women are more vulnerable to drug-induced QT prolongation, and similarly, hiPSC-CMs from women are more sensitive to rate-corrected field potential duration (FPDc) prolongation and arrhythmia incidence induced by *I*_Kr_ blockers [124, 125]. Similar to the result obtained from healthy human hearts [72], male-origin hiPSC-CMs displayed *KCNE1* upregulation [125], resulting in a greater response against *I*_Ks_ blockers [124, 125]. In addition, estradiol administration increases FPD in female-origin hiPSC-CMs, whereas testosterone shortens FPD/APD in male-origin hiPSC-CMs [124, 126], consistent with clinical observations in which the QT_C_ interval is shortened in males after puberty [29].

In conclusion, the intrinsic properties based on sex reveal fundamentally different responses in male and female-derived cells, making the hiPSC-CMs a useful model to study the mechanisms of sex differences and they help in predicting drug-induced arrhythmias in men and women. Furthermore, hiPSC-CMs can be maintained in a culture for a long time, making hiPSC-CMs a suitable model to study both the acute and the chronic effects of sex hormones. Sex-specific hiPSC-CMs enable us to control the environmental variables to study the genetic, epigenetic, and/or hormonal variables. Additionally, experimental variables such as the timing and dosage of hormonal application can also be optimized. Therefore, the development of male and female cardiac models helps achieve the goal of personalized medicine based on innovations in human stem cell technology without ethical constraints.

## Conclusions 

Both animal and human studies demonstrate that sex differences exist and range from gene expression to cardiac physiology. The presentation and progression of different cardiac diseases also differ in sex; men and women with cardiac diseases having the same gene mutations often have different clinical outcomes. Arrhythmia incidence is also different between men and women, and female sex itself is an independent risk factor a special ventricular arrhythmia, TdP, due to both genetic and acquired LQTS. Sex differences are an important factor to be considered when analyzing symptoms, searching for diagnosis, and optimizing the treatment for cardiac diseases.

Sex-specific differences in CMs and cardiac functions are most prominent after puberty demonstrating that sex hormones play an important role for causing such differences. However, detailed mechanisms underlying these sex differences are still unclear. In many studies, sex is not considered at all, causing misinterpretation of the actual results. Increasing the number of female participants in all types of studies, including animal experiments as well as clinical trials, and incorporating the sex as a factor in the analysis are needed to broaden our understanding of sex differences. Furthermore, endogenous sex hormone levels also change with age in both men and women. Therefore, experimental studies should incorporate not only sex, but also the sex hormone levels.

Currently, the data on sex-based electrophysiological and pharmacological responses from human-based models are limited and most of our understanding is still based on animal models. Although animal studies have delivered important information about sex differences and drug responses, such results cannot be directly translated in humans, and many drug therapies have still poorer outcomes in clinical studies than what was expected in animal studies [9]. One of the main reasons for this discrepancy is that cardiac physiology in animals differs from that in humans. Studies about sex-specific differences of new drugs should be performed in the human-based models with iPSC-derived male and female CMs. In addition, the sex differences should be consolidated into all phases of drug development to introduce safe and efficient drug treatment. Studies involving humans are expensive and can pose participants at an increased risk for side effects. Human-based heart biopsies are also limited, yielding a narrower human-based platform for research. Recent advances in hiPSC-CMs offer a robust human-based platform not only for a large drug screening, but also for studying the mechanisms of sex differences at cellular and tissue levels.

## Data Availability

Not applicable.

## Abbreviations

AP: Action potential
APA: Action potential amplitude
APD: Action potential duration
Ca^2+^: Calcium
cAMP: Cyclic adenosine monophosphate
CMs: Cardiomyocytes
dV/dt: Upstroke velocity
ECC: Excitation–contraction coupling
ECG: Electrocardiograph
FPDc: Rate-corrected field potential duration
HF: Heart failure
hiPSC-CMs: Human induced pluripotent stem cell derived cardiomyocytes
*I*_CaL_: L-type calcium current
*I*_K1_: Inward rectifier potassium current
*I*_Kr_: Rapid delayed rectifier potassium current
*I*_Ks_: Slow delayed rectifier potassium current
*I*_Kur_: Ultra-rapid delayed rectifier potassium current
*I*_Na_: Sodium current
*I*_to_: Transient outward potassium current
JT_C_: Rate-corrected JT interval
K^+^: Potassium
LQTS: Long QT syndrome
LVEF: Left ventricle ejection fraction
Na^+^: Sodium
NCX: Sodium calcium exchanger
PKA: Protein kinase A
PLB: Phospholamban
QT_C_: Rate-corrected QT intervals
RMP: Resting membrane potential
RyR2: Ryanodine receptor 2
SERCA: Sarcoplasmic reticulum Ca^2+^-ATPase
SR: Sarcoplasmic reticulum
TdP: Torsades de pointes
TWA: T-wave amplitude
VF: Ventricular fibrillation
